# Adapting to the Cold: A Role for Endogenous Fibroblast Growth Factor 21 in Thermoregulation?

**DOI:** 10.3389/fendo.2020.00389

**Published:** 2020-07-02

**Authors:** Marlou Klein Hazebroek, Susanne Keipert

**Affiliations:** Department of Molecular Biosciences, The Wenner-Gren Institute, Stockholm University, Stockholm, Sweden

**Keywords:** cold exposure, brown adipose tissue, beige adipose tissue, uncoupling protein 1, endocrine signaling, low protein diet, energy metabolism

## Abstract

Fibroblast growth factor 21 (FGF21) is in biomedical focus as a treatment option for metabolic diseases, given that administration improves metabolism in mice and humans. The metabolic effects of exogenous FGF21 administration are well-characterized, but the physiological role of endogenous FGF21 has not been fully understood yet. Despite cold-induced FGF21 expression and increased circulating levels in some studies, which co-occur with brown fat thermogenesis, recent studies in cold-acclimated mice demonstrate the dispensability of FGF21 for maintenance of body temperature, thereby questioning FGF21's role for thermogenesis. Here we discuss the evidence either supporting or opposing the role of endogenous FGF21 for thermogenesis based on the current literature. FGF21, secreted by brown fat or liver, is likely not required for energy homeostasis in the cold, but the nutritional conditions could modulate the interaction between FGF21, energy metabolism, and thermoregulation.

## Introduction

The ability to combat cold stress is crucial for the survival of mammals. Especially in small rodents and human infants, brown adipose tissue (BAT) and its unique protein uncoupling protein 1 (UCP1) play an important role in the maintenance of core body temperature (1). When activated, UCP1 uncouples mitochondrial respiration from ATP production, resulting in the direct dissipation of oxidative energy as heat (2). The expression of UCP1 is not restricted to BAT. UCP1 can be recruited in white adipose tissue (WAT), classifying it as beige adipose tissue, through various stimuli, i.e., cold exposure, beta-adrenergic stimulation, and various peripheral signals. One of these peripheral signals is endocrine-acting metabolic regulator fibroblast growth factor 21 (FGF21).

**Figure 1:**
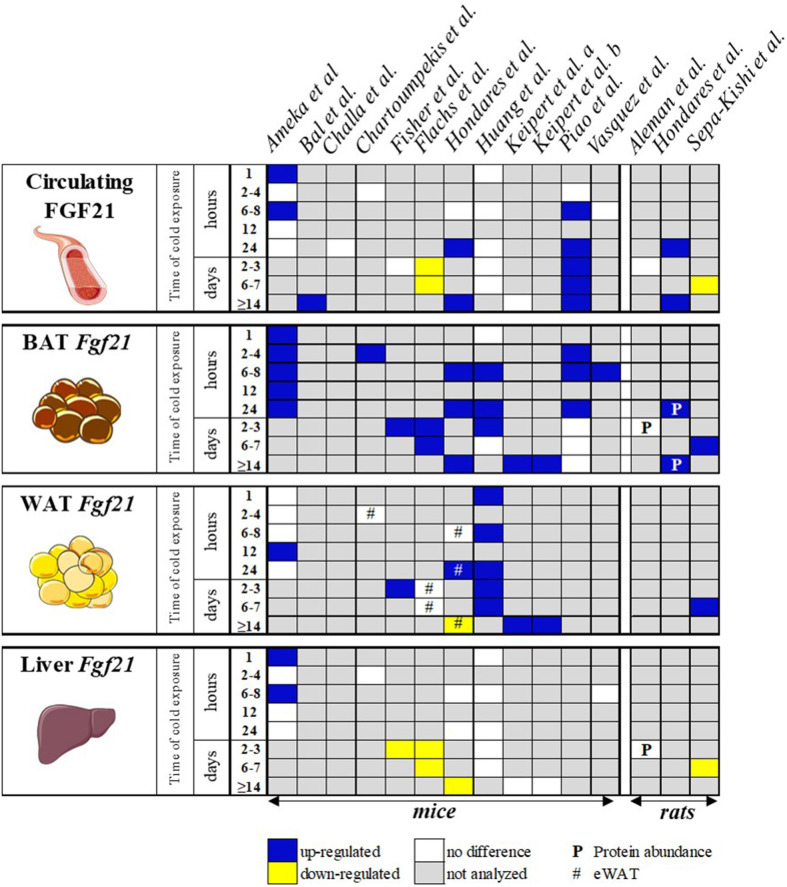
Schematic overview of publications investigating the effect of cold exposure on circulating FGF21 and *Fgf21* expression levels in various tissues of the WT control mice. Blue box, FGF21 was up-regulated; yellow box, FGF21 was down-regulated; white box, FGF21 not differently regulated *vs*. control; gray box, not analyzed. The data represent circulating levels and mRNA expression, unless otherwise specified. WAT, white adipose tissue (subcutaneous unless otherwise stated); BAT, brown adipose tissue. Keipert et al. a (13), b (14). Parts of this cartoon were created using Servier Medical Art (http://smart.servier.com).

It has been well-documented that the pharmacological administration of FGF21 and its analogs improves the metabolic profiles in mice and humans by decreasing adiposity, serum lipids, and blood glucose levels (3–7). Furthermore, the administration of FGF21 increases energy expenditure (7) and potently stimulates beige adipose tissue (8, 9). Its efficacy in humans promoted the interest in FGF21 as a promising therapeutic molecule for various metabolic diseases (e.g., obesity, type 2 diabetes, and non-alcoholic fatty liver disease). Therefore, it is not surprising that several clinical trials using FGF21 analogs have already reached phase 2 (10, 11) and are under investigation to enter phase 3 (12). On the other hand, the role of endogenous FGF21 is less established. Endogenous FGF21 is regulated upon cold exposure in mice (see, for an overview of references, Figure 1), and a growing number of publications indicate the regulation of circulating FGF21 in response to cold in humans (15–17).

Clarifying the underlying mechanisms of changes in endogenous levels upon cold is challenging because FGF21 can be secreted from various tissues, contributing to the serum concentration (18). So far, verifying the origin of FGF21 secretion in humans has been complicated. Although cold exposure is known to stimulate FGF21 expression in both humans and animal models, its metabolic consequence in thermogenically competent adipose tissue during cold remains incompletely understood. In this review, we will concentrate on the physiological role of endogenous FGF21 upon cold exposure and FGF21's metabolic effects, focusing on its importance in thermoregulation and metabolic homeostasis.

## FGF21'S Tissue Specificity and Regulation

FGF21 and its relatives, FGF15/19 and FGF23, are atypical members of the FGF family based on their role as endocrine factors (19). After synthesis and secretion, FGF21 acts as a circulating hormone that binds to fibroblast growth factor receptors and cofactor β*-*Klotho to form a ternary complex, which is responsible for FGF21 specificity and required to induce signal transduction in target tissues (20–23). The liver has been considered as the major source of circulating FGF21 in adult mice (24). Besides the liver, however, FGF21 is also synthesized in several other tissues, including the pancreas (25), skeletal muscle (26), and adipose tissue (13, 27). Over the last years, the central nervous system and adipose tissue have emerged as the key target organs of FGF21 action in improving metabolism (3, 28–30). For example, the insulin-sensitizing effects of FGF21 in mice are primarily regulated by adipose tissue (28), whereas the effects on energy expenditure and weight loss are mainly regulated centrally (31). The pharmacological administration of native FGF21 in mouse and human studies induces thermogenic gene expression in WAT and BAT, for example, *Ucp1* and *Dio2* (8, 9, 16). The induction of thermogenic genes can be a direct effect on adipose tissue (9) as well as indirect by the central nervous system (31, 32). FGF21 administration enhances the sympathetic outflow toward adipose tissue, possibly involving the induction of corticotropin-releasing hormone (31), which in turn activates the β-adrenergic-cAMP-mediated pathway, thereby turning on *Ucp1* expression. Furthermore, Fgf21 mRNA expression itself is induced in adipose tissue in response to stimulation with β-adrenergic agonists [e.g., isoproterenol; (9, 33)]. Although the pharmacological administration of FGF21 and ß-adrenergic agonists indicates a function of FGF21 in thermoregulation, the question on the role of endogenous FGF21 upon cold exposure remains unanswered.

## Is Circulating FGF21 an Endocrine Metabolic Regulator Upon Cold Exposure?

A study performed in neonatal mouse pups was the first to suggest an endocrine role of endogenous FGF21 in thermoregulation (34). Here the consumption of maternal milk (i.e., initial lipid intake) triggered liver *Fgf21* expression in newborn mice, leading to an increase in circulating FGF21 levels. Notably, the rise in FGF21 plasma levels parallels the thermogenic gene expression in neonatal BAT after birth. Given these results, the authors suggested a liver–FGF21–BAT axis, in which FGF21, secreted by the liver, contributes to the activation of BAT thermogenesis to defend the body temperature during early life. Although the mice were not housed at 4°C in this study, it inspired the idea that a liver–FGF21–BAT axis might also be present in thermogenesis in later life. Since then, many studies attempted to uncover the possible endocrine role of endogenous FGF21 upon cold exposure in adult mice, with controversial results.

Numerous studies show that circulating FGF21 is upregulated in wild-type (WT) mice housed at 4–6°C (34–38), while others did not observe this induction (9, 13, 33, 39–41) but instead report on a decrease [(42); Figure 1]. The inconsistencies between the studies may be explained by the differences in animal model or experimental design. However, a summary of the published data from 13 mouse and three rat studies (Table 1) reveals that gender, age, or genetic background cannot explain the differences as most of the studies used adult male mice on a C57BL/6J background or male Wistar rats. Furthermore, the housing temperature before cold treatment and the duration of the cold treatment seem to have no consistent effect on FGF21 secretion (Table 1 and Figure 1). In addition to the ambiguity of whether circulating FGF21 increases upon cold treatment, a closer inspection of the publications that observe an increase shows that there are inconsistencies concerning the tissue responsible for the secretion. Two main tissues arise, BAT and liver, which we will now discuss individually.

**Table 1:** Overview of experimental designs and study specifics of publications on FGF21 and cold exposure at 4–8°C.

**References**	**Study design (cold exposure)**	**Species and background**	**Genotype**	**Diet**	**Age at start (weeks)**	**Ambient temperature (*t* = 0)**
Ameka et al. (35)	30 min, 1, 2, 6, 12, and 24 h at 4°C	Mice (C57Bl/6J)	FGF21^fl/fl^ × albumin-Cre FGF21 ^fl/fl^ × adiponectin-Cre KLB ^fl/fl^ × adiponectin-Cre +corresponding wild-type (WT) control	Standard chow	12	30°C (72 h)
Bal et al. (36)	5 d at 16°C → 15 d at 4°C	Mice (C57BL/6J)	WT	Regular rodent diet	12	29°C
Challa et al. (39)	1 d at 8°C	Mice (C57/B6N)	WT, UCP1-DTR-eGFP	Standard chow	8–12	23°C
Chartoumpekis et al. (33)	4 h at 4°C	Mice (C57Bl/6J)	WT	Standard chow (fasted at 4°C)	24	22°C
Fisher et al. (9)	72 h at 5°C	Mice (C57Bl/6)	WT, FGF21 KO	Not stated	11	27°C (6 d)
Flachs et al. (42)	2 and 7 d at 6°C	Mice (C57Bl/6J*;* A/J mice)	WT	Standard chow	9	30°C (7 d)
Hill et al. (37)	6 h at 4°C	Mice (C57Bl/6J)	WT, FGF21 KO	Low-protein and control diet	12	23°C (10 d)
Hondares et al. (27)	6 and 24 h; 30 d at 4°C	Mice (Swiss)	WT (Swiss mice), PPARa-null (129S4/SvJae-Pparatm1Gonz/J)	Not stated	Adult	29°C
Huang et al. (40)	1, 6, 24 h and 3, 6 d at 6°C	Mice (C57Bl/6J)	FGF21^lox/lox^ × aP2-Cre FGF21^lox/lox^ × albumin-Cre KLB^lox/lox^ × adiponectin-Cre +corresponding WT control	Standard chow	12	30°C (2 wks)
Keipert et al. (13)	~3 wks at 18°C → 4 wks at 5°C	Mice (C57Bl/6J)	WT, UCP1-KO	Standard chow	~11	30°C (~11 wks)
Keipert et al. (14)	2 wks at 18°C → 3 wks at 5°C	Mice (C57Bl/6J)	WT, FGF21 KO, UCP1-KO, and UCP1/FGF21 double KO	Standard chow	~11	30°C (~11 wks)
Piao et al. (38)	2, 8, and 24 h; 3, 7, and 14 d at 4°C	Mice (C57Bl/6J)	WT	Standard chow	11	Not stated
Vázquez et al. (41)	6 h at 4°C	Mice (C57Bl/6J)	WT, heterozygote tyrosine hydroxylase KO (Th+/–)	Standard chow	8–12	29°C (1 wk)
Alemán et al. (43)	72 h at 4°C	Rats (Wistar)	WT	6, 20, and 50% protein diet (time restricted)	6	23°C
Hondares et al. (27)	24 h; 30 d at 4°C	Rats (Wistar)	WT	Not stated	Not stated	29°C (3 wks)
Sepa-Kishi et al. (44)	7 d at 4°C	Rats (Wistar)	WT	Standard chow	Not stated	22°C

*All studies used male mice and rats. h, hour(s); d, day(s); wk(s), week(s); → “followed by”*.

## Brown Adipose Tissue as the Source for Circulating FGF21

Hondares et al. (27) were the first to show increased circulating FGF21 levels upon cold exposure in adult mice (27). After 6 h at 4°C, the circulating FGF21 levels were not different compared to those of mice kept at 30°C, yet the mice showed increased levels after 24 h and 30 days at 4°C. A gene expression analysis suggested BAT as an FGF21 source, as reflected in induced mRNA levels. In contrast, *Fgf21* mRNA in the liver remained unchanged (6 h) or even decreased upon long-term cold exposure (24 h). In the same study, but using rats, *in vivo* arteriovenous differences in plasma FGF21 across interscapular BAT were measured, confirming the release of FGF21 from BAT (27). In 2018, others followed up on these findings, showing that the circulating FGF21 levels increase throughout 8 h to 14 days of cold treatment period (38). The authors claim BAT to be the source of increased FGF21 as this was associated with an early induction of *Fgf21* gene expression in this tissue, which declines after 7 days. When interpreting these data, we may bear in mind that no further gene expression data of other potential FGF21 secreting tissues were included, as well as the lack of specific knock-out models. Thus, it is difficult to determine the tissue responsible for the increase in circulating FGF21. However, Piao et al. (38) showed an important role of circulating FGF21 action in the induction of WAT browning during cold acclimation (38). Neither centrally nor peripherally administered FGF21 initiated the induction of beige fat in mice lacking β-adrenoceptors, demonstrating that an intact adrenergic system is necessary for FGF21 action (31, 32). These data suggest FGF21 signals via the brain to activate the sympathetic nervous system and induce adipose tissue thermogenesis.

In UCP1 knockout (KO) mice, a model that lacks classical, UCP1-mediated BAT thermogenesis (45, 46), BAT becomes a potential source of circulating FGF21 upon long-term cold exposure. Gene expression as well as *ex vivo* tissue secretion assays confirm BAT but exclude muscle, WAT, and liver as sources of circulating FGF21 in these mice (13). Interestingly, this study could not confirm the previous observations on the release of FGF21 from BAT in WT mice (27), which were exposed to long-term cold. It has been frequently speculated that other heat sources (e.g., creatine-driven substrate cycling and PM20D1-regulated mitochondrial uncoupling) are alternatively activated to protect the body temperature (47, 48), possibly in other tissue sites such as subcutaneous WAT (sWAT). Consistent with the literature that claims the induction of browning by FGF21 (8, 9), pronounced browning features in sWAT of UCP1-KO mice, but not in WT mice, were observed (13). Thus, the significant release of FGF21 from non-functional BAT suggests the possibility of an endocrine crosstalk between BAT and other tissues to coordinate a systemic adaptive thermogenesis response in UCP1-KO mice.

To consolidate FGF21 dependency, UCP1-FGF21 double knockout (dKO) mice were created and characterized (14). Unexpectedly, the dKO mice displayed identical energy expenditure, browning of WAT, and defense of body temperature during long-term cold exposure as their single knockout, UCP1-KO, counterparts. This observation strongly suggests that the increases of FGF21 serum levels in UCP1-KO mice have no major impact on thermoregulatory capacity. Thus, one can highlight that FGF21 *per se* is not required for the defense of body temperature despite a strong induction of FGF21 in various adipose tissue depots during prolonged cold exposure (14).

## Liver as the Source for Circulating FGF21

The contribution of different tissues to circulating FGF21 in cold-induced thermogenesis was further dissected in a study that used adipose- and liver-specific knockout mouse models and cold exposure ranging from 1 h up to 6 days (40). Interestingly, the authors did not observe an increase in circulating FGF21 levels and therefore excluded an endocrine role for FGF21. In line with other studies (Figure 1), no increase of *Fgf21* mRNA in the liver was detected upon cold exposure, and the adult liver-specific FGF21-KO mice did not show impairment in thermoregulation upon cold exposure. However, *Fgf21* mRNA and protein are highly cold-induced in both BAT and WAT. Furthermore, the adipose tissue-specific FGF21-KO mice show reduced body temperature and recruitment of beige adipocytes in the early phase of cold exposure (40). This impairment of cold-induced adaptive thermogenesis suggests an autocrine thermoregulatory role of FGF21 in WAT and proposes the induction of beige adipocytes as an active player in thermogenesis.

Strongly opposing these results are the data of Ameka et al. (35). While both studies excluded BAT as a source of FGF21 upon cold exposure, here the authors highlight the liver as the source of circulating FGF21 in response to short-term cold exposure. In direct contrast to Huang et al. (40), the liver-specific FGF21-KO mice display decreased body temperatures upon short-term cold exposure, whereas the adipose-specific FGF21-KO mice do not. A liver FGF21–brain–BAT axis was highlighted by showing reduced sympathetic nerve activity to BAT in liver-specific FGF21-KO mice as well as in WT mice with pharmacologically inhibited FGF21 signaling in the brain (35).

An explanation for the study differences on the adipose tissue mouse model may reside in the use of the Cre mouse model. While Ameka et al. used the adiponectin-Cre mouse, Huang et al. used the aP2-Cre transgenic mouse, which expresses Cre recombinase not exclusively in adipose tissue (17). However, the differences found in the liver-specific KO mouse model remain puzzling.

## Is There an Auto-Paracrine Role of Thermogenic Adipose FGF21 Upon Cold Exposure?

A general theme that crystalizes from nearly all published data is the induction of *Fgf21* mRNA in BAT upon cold exposure (Figure 1), indicating a possible auto- paracrine role of FGF21 when the circulating levels remain unchanged. The inducing signaling cascade for *Fgf21* expression is almost identical as that found for UCP1 regulation: cyclic-AMP mediates the activation of protein kinase A and p38 MAPK, which activates transcription factor 2 for transactivation of the *Ucp1* and *Fgf21* gene promoters (9, 27, 49), suggesting that adipose FGF21 is a downstream effector of sympathetic nerve activation. Surprisingly, however, the studies showing decreased body temperatures during cold exposure of FGF21-KO mice, including global and liver- and adipose-tissue specific knockouts, did not detect impairment of BAT thermogenic gene expression after cold exposure compared to WT mice (9, 35, 40). Furthermore, no differences in UCP1 protein content, adipose morphology, or mitochondrial content were seen (40), suggesting that BAT is not the main driver for body temperature reductions in FGF21-KO mice. Two of these three studies, which do not detect any increase in circulating FGF21, instead found reduced thermogenic gene expression in WAT of FGF21-KO mice (9, 40), lower UCP1 protein levels, and less beige fat cells and mitochondrial content (40). These findings, once more, strongly support the auto-paracrine control of FGF21 over the “browning” of WAT, which in turn may contribute to thermogenesis.

During long-term cold exposure, however, no differences in thermogenic gene and protein expression, as well as in adipocyte morphology, were found in the BAT and sWAT of FGF21-KO and WT mice (14). The interesting differences in BAT morphology between UCP1-KO and UCP1-FGF21 dKO mice, measured as a drastically reduced number of lipid droplets in the double-knockout mice, are so far unexplored. The comparison of global BAT transcriptomes in this study revealed FGF21 as one of the top regulated genes, followed by genes involved in the feedback regulation of FGF21 signaling (e.g., *Dusp4* and *Spry4*). These observations strongly suggest a bioactivity of endogenous FGF21 *in vivo* and may be interpreted as auto-paracrine effects of supra-stimulated FGF21 function in UCP1-KO mice, possibly related to lipid metabolism. Notably, the UCP1-KO mice show reduced survival rates when weaned and raised at 21°C, which represents a temperature of moderate cold stress for mice, thereby highlighting the important role of UCP1 in thermoregulation. In contrast, the homozygous FGF21-KO mice, weaned and raised at 21°C, do not show reduced survival rates or reduced body weight compared to their WT littermates (50, 51), thereby excluding a major role of FGF21 on the protection of body temperature during early life development.

## The Impact of Diet and Mild Cold-Induced Endogenous FGF21 on Thermoregulation

Protein restriction in food is a potent inducer of FGF21 secretion from the liver into the circulation (52, 53). Alemán et al. (43) studied the interaction of temperature effects and the amount of dietary protein on thermogenic adipose tissue in rats (43). As expected, a low-protein diet led to an increase in liver *Fgf21* expression and circulating FGF21, as well as to an increase in oxygen consumption rates at 23 and 4°C, compared to an adequate-protein-content diet. Interestingly, an increase of UCP1 protein concentration was not seen in BAT but in sWAT of low-protein-fed rats kept at 23 and 4°C. The authors speculated that low-protein diets stimulate energy expenditure via FGF21-induced UCP1 protein expression in WAT. In line with this is the induction of *Ucp1* gene expression only in sWAT, and not in BAT or epididymal WAT with low-protein diets, which disappears in liver-specific FGF21-KO mice (54). Moreover, no increase in energy expenditure was seen in UCP1-KO mice after low-protein-diet feeding (37). In this study, it was further investigated whether the animals with high circulating FGF21 can cope better, with an acute cold stimulus, during low-protein-diet feeding. The increase of endogenous FGF21 levels in WT mice fed a low protein diet, however, had no effect on the acute thermogenic responses to cold, measured as body temperature (37). Therefore, we may conclude that albeit the increased circulating liver-derived FGF21 levels induce *Ucp1* gene expression in sWAT, this is not required for physiological protection from acute cold stress.

As mentioned previously, neither UCP1 nor FGF21 is required during adulthood to defend the body temperature from long-term cold exposure (14). It seems that an ambient temperature of 5°C for several weeks overrules FGF21 action, leading to FGF21-independent browning of WAT in UCP1-KO mice. Surprisingly, some of our newest data highlight mild cold-induced endogenous FGF21 as the primary metabolic regulator of obesity resistance, possibly mediated via WAT browning in UCP1-KO mice (55). How could the contradiction of the impact of FGF21 on browning of WAT between these studies be explained? There are two important differences in the study setup. In the most recent study, UCP1-KO mice were transferred from 30°C to mild cold (23°C) instead of 4°C and the diet was switched from chow to high-fat diet. While mild cold exposure is essential to trigger FGF21 release in UCP1-KO mice, FGF21 only unfolds its anti-obesity effect in combination with overnutrition in the form of high-fat diets. This rationale is coherent with the pharmacology of exogenous FGF21, which does not reduce body weight in lean mice, but only in obese mice, and partly requires UCP1-independent thermogenesis for its beneficial metabolic effects (6, 56). This hypothesis is further strengthened by the requirement of increased endogenous FGF21 for the protection from HFD-induced body weight gain in different other mouse models (57, 58).

## Summary and Conclusion

Ever since endogenous FGF21 has been associated to the expression of thermogenic genes in BAT of neonatal pups, researchers have been investigating FGF21's role for survival in the cold. Unfortunately, the results are, to some extent, contradictory. On one hand, observations suggest higher circulating FGF21 levels upon cold exposure, while others report no changes or even decreased levels. When analyzing the studies that report increased levels, two tissue sites emerge as potential secretors: one being BAT, the other being the liver. Despite using sophisticated methods such as arteriovenous differences in plasma FGF21 across tissues and tissue-specific FGF21-KO mouse models, some of the results remain opposing, thereby revealing no unambiguous picture. While these contradictions could highlight the complexity of endogenous FGF21 action, one may also conclude that there is no crucial role of endocrine-acting FGF21 during cold exposure. Common in all studies is the increase of *Fgf21* mRNA expression in BAT during cold exposure, raising the possibility of auto- or paracrine signaling. However, the direct comparison of FGF21-KO mice and WT mice did not reveal cold-induced morphological or molecular differences in BAT compared to WT mice, despite a high induction of *Fgf21* gene expression, suggesting a minor auto- paracrine role of FGF21 in BAT. The current literature instead predominantly supports an indirect as well as a direct impact of FGF21 on WAT in the early cold response, as well as during the combination of mild cold and nutritional composition. Given the sensitivity of FGF21 regulation to the nutritional composition of food and its pronounced effects on browning of WAT, the impact of FGF21 on thermoregulation during mild cold cannot be fully excluded. Further research is required to unravel the underlying molecular mechanisms that may be beneficial for the understanding of human obesity and metabolic diseases.

## Author Contributions

MK and SK wrote this review article in concert, read, and approved the submitted version. All authors contributed to the article and approved the submitted version.

## Conflict of Interest

The authors declare that the research was conducted in the absence of any commercial or financial relationships that could be construed as a potential conflict of interest.

## References

[1] Cannon B Nedergaard J. Brown adipose tissue: function and physiological significance. Physiol Rev. (2004) 84:277–359. 10.1152/physrev.00015.2003 14715917

[2] Nicholls DG Locke RM. Thermogenic mechanisms in brown fat. Physiol Rev. (1984) 64:1–64. 10.1152/physrev.1984.64.1.1 6320232

[3] Coskun T Bina HA Schneider MA Dunbar JD Hu CC Chen Y. Fibroblast growth factor 21 corrects obesity in mice. Endocrinology. (2008) 149:6018–27. 10.1210/en.2008-0816 18687777

[4] Gaich G Chien JY Fu H Glass LC Deeg MA Holland WL. The effects of LY2405319, an FGF21 analog, in obese human subjects with type 2 diabetes. Cell Metab. (2013) 18:333–40. 10.1016/j.cmet.2013.08.005 24011069

[5] Talukdar S Zhou Y Li D Rossulek M Dong J Somayaji V. A long-acting FGF21 molecule, PF-05231023, decreases body weight and improves lipid profile in non-human primates and type 2 diabetic subjects. Cell Metab. (2016) 23:427–40. 10.1016/j.cmet.2016.02.001 26959184

[6] Véniant MM Komorowski R Chen P Stanislaus S Winters K Hager T. Long-acting FGF21 has enhanced efficacy in diet-induced obese mice and in obese rhesus monkeys. Endocrinology. (2012) 153:4192–203. 10.1210/en.2012-1211 22798348

[7] Xu J Lloyd DJ Hale C Stanislaus S Chen M Sivits G. Fibroblast growth factor 21 reverses hepatic steatosis, increases energy expenditure, and improves insulin sensitivity in diet-induced obese mice. Diabetes. (2009) 58:250–9. 10.2337/db08-0392 18840786 PMC2606881

[8] Emanuelli B Vienberg SG Smyth G Cheng C Stanford KI Arumugam M. Interplay between FGF21 and insulin action in the liver regulates metabolism. J Clin Invest. (2014) 124:515–27. 10.1172/JCI67353 24401271 PMC3904602

[9] Fisher FM Kleiner S Douris N Fox EC Mepani RJ Verdeguer F. FGF21 regulates PGC-1α and browning of white adipose tissues in adaptive thermogenesis. Genes Dev. (2012) 26:271–81. 10.1101/gad.177857.111 22302939 PMC3278894

[10] Charles ED Neuschwander-Tetri BA Frias JP Kundu S Luo Y Tirucherai GS. Pegbelfermin (BMS-986036), PEGylated FGF21, in patients with obesity and type 2 diabetes: results from a randomized phase 2 study. Obesity. (2019) 27:41–9. 10.1002/oby.22344 30520566 PMC6587787

[11] Sanyal A Charles ED Neuschwander-Tetri BA Loomba R Harrison SA Abdelmalek MF. Pegbelfermin (BMS-986036), a PEGylated fibroblast growth factor 21 analogue, in patients with non-alcoholic steatohepatitis: a randomised, double-blind, placebo-controlled, phase 2a trial. Lancet. (2018) 392:2705–17. 10.1016/S0140-6736(18)31785-9 30554783

[12] Verzijl CRC Peppel IPVD Struik D Jonker JW. Pegbelfermin (BMS-986036): an investigational PEGylated fibroblast growth factor 21 analogue for the treatment of nonalcoholic steatohepatitis. Expert Opin Investig Drugs. (2020) 29:125–33. 10.1080/13543784.2020.1708898 31899984

[13] Keipert S Kutschke M Lamp D Brachthäuser L Neff F Meyer CW. Genetic disruption of uncoupling protein 1 in mice renders brown adipose tissue a significant source of FGF21 secretion. Mol Metab. (2015) 4:537–42. 10.1016/j.molmet.2015.04.006 26137441 PMC4481421

[14] Keipert S Kutschke M Ost M Schwarzmayr T van Schothorst EM Lamp D Long-term cold adaptation does not require FGF21 or UCP1. Cell Metab. (2017) 26:437–46.e5. 10.1016/j.cmet.2017.07.016 28768181

[15] Hanssen MJW Broeders E Samms RJ Vosselman MJ van der Lans AAJJ Cheng CC. Serum FGF21 levels are associated with brown adipose tissue activity in humans. Sci Rep. (2015) 5:10275. 10.1038/srep10275 25985218 PMC4434994

[16] Lee P Linderman JD Smith S Brychta RJ Wang J Idelson C. Irisin and FGF21 are cold-induced endocrine activators of brown fat function in humans. Cell Metab. (2014) 19:302–9. 10.1016/j.cmet.2013.12.017 24506871 PMC7647184

[17] Lee P Brychta RJ Linderman J Smith S Chen KY Celi FS. Mild cold exposure modulates fibroblast growth factor 21 (FGF21) diurnal rhythm in humans: relationship between FGF21 levels, lipolysis, and cold-induced thermogenesis. J Clin Endocrinol Metab. (2013) 98:E98–102. 10.1210/jc.2012-3107 23150685 PMC3537100

[18] Fisher FM Maratos-Flier E. Understanding the physiology of FGF21. Annu Rev Physiol. (2016) 78:223–41. 10.1146/annurev-physiol-021115-105339 26654352

[19] Itoh N. Hormone-like (endocrine) Fgfs: their evolutionary history and roles in development, metabolism, and disease. Cell Tissue Res. (2010) 342:1–11. 10.1007/s00441-010-1024-2 20730630 PMC2948652

[20] Adams AC Cheng CC Coskun T Kharitonenkov A. FGF21 requires βklotho to act *in vivo*. PLoS ONE. (2012) 7:e49977. 10.1371/journal.pone.0049977 23209629 PMC3507945

[21] Kharitonenkov A Dunbar JD Bina HA Bright S Moyers JS Zhang C. FGF-21/FGF-21 receptor interaction and activation is determined by βKlotho. J Cell Physiol. (2008) 215:1–7. 10.1002/jcp.21357 18064602

[22] Kurosu H Choi M Ogawa Y Dickson AS Goetz R Eliseenkova AV. Tissue-specific expression of betaKlotho and fibroblast growth factor (FGF) receptor isoforms determines metabolic activity of FGF19 and FGF21. J Biol Chem. (2007) 282:26687–95. 10.1074/jbc.M704165200 17623664 PMC2496965

[23] Ogawa Y Kurosu H Yamamoto M Nandi A Rosenblatt KP Goetz R. BetaKlotho is required for metabolic activity of fibroblast growth factor 21. Proc Natl Acad Sci USA. (2007) 104:7432–7. 10.1073/pnas.0701600104 17452648 PMC1855074

[24] Nishimura T Nakatake Y Konishi M Itoh N. Identification of a novel Fgf, Fgf-21, preferentially expressed in the liver. Biochim Biophys Acta. (2000) 1492:203–6. 10.1016/S0167-4781(00)00067-1 10858549

[25] Coate KC Hernandez G Thorne CA Sun S Le TDV Vale K. FGF21 is an exocrine pancreas secretagogue. Cell Metab. (2017) 25:472–80. 10.1016/j.cmet.2016.12.004 28089565 PMC5299054

[26] Keipert S Ost M Johann K Imber F Jastroch M van Schothorst EM. Skeletal muscle mitochondrial uncoupling drives endocrine cross-talk through the induction of FGF21 as a myokine. Am J Physiol Endocrinol Metab. (2014) 306:E469–482. 10.1152/ajpendo.00330.2013 24347058

[27] Hondares E Iglesias R Giralt A Gonzalez FJ Giralt M Mampel T. Thermogenic activation induces FGF21 expression and release in brown adipose tissue. J Biol Chem. (2011) 286:12983–90. 10.1074/jbc.M110.215889 21317437 PMC3075644

[28] BonDurant LD Ameka M Naber MC Markan KR Idiga SO Acevedo MR. FGF21 regulates metabolism through adipose-dependent and -independent mechanisms. Cell Metab. (2017) 25:935–944.e4. 10.1016/j.cmet.2017.03.005 28380381 PMC5494834

[29] Bookout AL de Groot MHM Owen BM Lee S Gautron L Lawrence HL. FGF21 regulates metabolism and circadian behavior by acting on the nervous system. Nat Med. (2013) 19:1147–52. 10.1038/nm.3249 23933984 PMC3769420

[30] Song P Zechner C Hernandez G Cánovas J Xie Y Sondhi V. The hormone FGF21 stimulates water drinking in response to ketogenic diet and alcohol. Cell Metab. (2018) 27:1338–1347.e4. 10.1016/j.cmet.2018.04.001 29657029 PMC5990458

[31] Owen BM Ding X Morgan DA Coate KC Bookout AL Rahmouni K. FGF21 acts centrally to induce sympathetic nerve activity, energy expenditure, and weight loss. Cell Metab. (2014) 20:670–7. 10.1016/j.cmet.2014.07.012 25130400 PMC4192037

[32] Douris N Stevanovic DM Fisher FM Cisu TI Chee MJ Nguyen NL. Central fibroblast growth factor 21 browns white fat via sympathetic action in male mice. Endocrinology. (2015) 156:2470–81. 10.1210/en.2014-2001 25924103 PMC4475718

[33] Chartoumpekis DV Habeos IG Ziros PG Psyrogiannis AI Kyriazopoulou VE Papavassiliou AG. Brown adipose tissue responds to cold and adrenergic stimulation by induction of FGF21. Mol Med Camb Mass. (2011) 17:736–40. 10.2119/molmed.2011.00075 21373720 PMC3146611

[34] Hondares E Rosell M Gonzalez FJ Giralt M Iglesias R Villarroya F. Hepatic FGF21 expression is induced at birth via PPARα in response to milk intake and contributes to thermogenic activation of neonatal brown fat. Cell Metab. (2010) 11:206–12. 10.1016/j.cmet.2010.02.001 20197053 PMC2847690

[35] Ameka M Markan KR Morgan DA BonDurant LD Idiga SO Naber MC. Liver derived FGF21 maintains core body temperature during acute cold exposure. Sci Rep. (2019) 9:630. 10.1038/s41598-018-37198-y 30679672 PMC6345819

[36] Bal NC Maurya SK Pani S Sethy C Banerjee A Das S. Mild cold induced thermogenesis: are BAT and skeletal muscle synergistic partners? Biosci Rep. (2017) 37:BSR20171087. 10.1042/BSR20171087 28831023 PMC5617911

[37] Hill CM Laeger T Albarado DC McDougal DH Berthoud H-R Münzberg H. Low protein-induced increases in FGF21 drive UCP1-dependent metabolic but not thermoregulatory endpoints. Sci Rep. (2017) 7:8209. 10.1038/s41598-017-07498-w 28811495 PMC5557875

[38] Piao Z Zhai B Jiang X Dong M Yan C Lin J. Reduced adiposity by compensatory WAT browning upon iBAT removal in mice. Biochem Biophys Res Commun. (2018) 501:807–13. 10.1016/j.bbrc.2018.05.089 29775611

[39] Challa TD Dapito DH Kulenkampff E Kiehlmann E Moser C Straub L. A genetic model to study the contribution of brown and brite adipocytes to metabolism. Cell Rep. (2020) 30:3424–3433.e4. 10.1016/j.celrep.2020.02.055 32160547

[40] Huang Z Zhong L Lee JTH Zhang J Wu D Geng L. The FGF21-CCL11 axis mediates beiging of white adipose tissues by coupling sympathetic nervous system to type 2 immunity. Cell Metab. (2017) 26:493–508.e4. 10.1016/j.cmet.2017.08.003 28844880

[41] Vázquez P Hernández-Sánchez C Escalona-Garrido C Pereira L Contreras C López M. Increased FGF21 in brown adipose tissue of tyrosine hydroxylase heterozygous mice: implications for cold adaptation. J Lipid Res. (2018) 59:2308–20. 10.1194/jlr.M085209 30352954 PMC6277155

[42] Flachs P Adamcova K Zouhar P Marques C Janovska P Viegas I Induction of lipogenesis in white fat during cold exposure in mice: link to lean phenotype. Int J Obes. (2017) 41:997 10.1038/ijo.2017.61 28584260

[43] Alemán G Castro AL Vigil-Martínez A Torre-Villalvazo I Díaz-Villaseñor A Noriega LG. Interaction between the amount of dietary protein and the environmental temperature on the expression of browning markers in adipose tissue of rats. Genes Nutr. (2019) 14:19. 10.1186/s12263-019-0642-x 31178938 PMC6549346

[44] Sepa-Kishi DM Ceddia RB. Circulating fibroblast growth factor 21 is reduced, whereas its production is increased in a fat depot-specific manner in cold-acclimated rats. Adipocyte. (2018) 7:238–47. 10.1080/21623945.2018.1504591 30059270 PMC6768246

[45] Enerbäck S Jacobsson A Simpson EM Guerra C Yamashita H Harper ME. Mice lacking mitochondrial uncoupling protein are cold-sensitive but not obese. Nature. (1997) 387:90–4. 10.1038/387090a0 9139827

[46] Golozoubova V Cannon B Nedergaard J. UCP1 is essential for adaptive adrenergic nonshivering thermogenesis. Am J Physiol Endocrinol Metab. (2006) 291:E350–7. 10.1152/ajpendo.00387.2005 16595854

[47] Kazak L Chouchani ET Jedrychowski MP Erickson BK Shinoda K Cohen P. A creatine-driven substrate cycle enhances energy expenditure and thermogenesis in beige fat. Cell. (2015) 163:643–55. 10.1016/j.cell.2015.09.035 26496606 PMC4656041

[48] Long JZ Svensson KJ Bateman LA Lin H Kamenecka T Lokurkar IA. The Secreted enzyme PM20D1 regulates lipidated amino acid uncouplers of mitochondria. Cell. (2016) 166:424–35. 10.1016/j.cell.2016.05.071 27374330 PMC4947008

[49] Cao W Daniel KW Robidoux J Puigserver P Medvedev AV Bai X. p38 mitogen-activated protein kinase is the central regulator of cyclic AMP-dependent transcription of the brown fat uncoupling protein 1 gene. Mol Cell Biol. (2004) 24:3057–67. 10.1128/mcb.24.7.3057-3067.2004 15024092 PMC371122

[50] Hotta Y Nakamura H Konishi M Murata Y Takagi H Matsumura S. Fibroblast growth factor 21 regulates lipolysis in white adipose tissue but is not required for ketogenesis and triglyceride clearance in liver. Endocrinology. (2009) 150:4625–33. 10.1210/en.2009-0119 19589869

[51] Potthoff MJ Inagaki T Satapati S Ding X He T Goetz R. FGF21 induces PGC-1α and regulates carbohydrate and fatty acid metabolism during the adaptive starvation response. Proc Natl Acad Sci USA. (2009) 106:10853–8. 10.1073/pnas.0904187106 19541642 PMC2705613

[52] Laeger T Henagan TM Albarado DC Redman LM Bray GA Noland RC. FGF21 is an endocrine signal of protein restriction. J Clin Invest. (2014) 124:3913–22. 10.1172/JCI74915 25133427 PMC4153701

[53] Maida A Zota A Sjøberg KA Schumacher J Sijmonsma TP Pfenninger A. A liver stress-endocrine nexus promotes metabolic integrity during dietary protein dilution. J Clin Invest. (2016) 126:3263–78. 10.1172/JCI85946 27548521 PMC5004939

[54] Pérez-Martí A Garcia-Guasch M Tresserra-Rimbau A Carrilho-Do-Rosário A Estruch R Salas-Salvadó J. A low-protein diet induces body weight loss and browning of subcutaneous white adipose tissue through enhanced expression of hepatic fibroblast growth factor 21 (FGF21). Mol Nutr Food Res. (2017) 61:1600725. 10.1002/mnfr.201600725 28078804

[55] Keipert S Lutter D Schroeder BO Brandt D Ståhlman M Schwarzmayr T. Endogenous FGF21-signaling controls paradoxical obesity resistance of UCP1-deficient mice. Nat Commun. (2020) 11:624. 10.1038/s41467-019-14069-2 32005798 PMC6994690

[56] Samms RJ Smith DP Cheng CC Antonellis PP Perfield JW Kharitonenkov A. Discrete aspects of FGF21 *in vivo* pharmacology do not require UCP1. Cell Rep. (2015) 11:991–9. 10.1016/j.celrep.2015.04.046 25956583

[57] Lynch L Hogan AE Duquette D Lester C Banks A LeClair K. iNKT cells induce FGF21 for thermogenesis and are required for maximal weight loss in GLP1 therapy. Cell Metab. (2016) 24:510–9. 10.1016/j.cmet.2016.08.003 27593966 PMC5061124

[58] Wall CE Whyte J Suh JM Fan W Collins B Liddle C. High-fat diet and FGF21 cooperatively promote aerobic thermogenesis in mtDNA mutator mice. Proc Natl Acad Sci USA. (2015) 112:8714–9. 10.1073/pnas.1509930112 26124126 PMC4507233

